# Only EBUS-Guided Mediastinal Lymph Node Cryobiopsy Enabled Immunotherapy in a Patient with Non-Small Cell Lung Cancer

**DOI:** 10.3390/jcm12062355

**Published:** 2023-03-17

**Authors:** Jürgen Hetzel, Laetitia A. Mauti, Jonas Winkler, Sabine Cardoso Almeida, Philip Jermann, Miklos Pless, Lukas Bubendorf, Peter Karl Bode, Maik Häntschel

**Affiliations:** 1 Department of Medical Oncology and Pneumology, Eberhard Karls University, 72076 Tübingen, Germany; 2 Department of Internal Medicine—Pneumology, Cantonal Hospital Winterthur, 8401 Winterthur, Switzerland; 3 Department of Internal Medicine—Medical Oncology, Cantonal Hospital Winterthur, 8401 Winterthur, Switzerland; 4 Institute of Pathology, Cantonal Hospital Winterthur, 8401 Winterthur, Switzerland; 5 Institute of Medical Genetics and Pathology, University Hospital Basel, University of Basel, 4031 Basel, Switzerland

**Keywords:** lung cancer, NSCLC, bronchoscopy, cryobiopsy, mediastinal lymph node biopsy

## Abstract

Personalized treatment of metastatic non-squamous non-small cell lung cancer (NSCLC) requires detailed molecular characterization of the tumour including detection of predictive driver mutations and programmed death ligand 1 (PD-L1) expression. Complete detection is influenced by the amount of tumour cells sampled as well as their quality. Different sampling techniques may be necessary to provide sufficient tumour material for comprehensive molecular characterization. Missing the detection of targetable molecular genetic aberrations would have a serious impact on the quality of life and prognosis of a patient. This case report highlights the importance of biopsy technique in a patient with NSCLC. Several procedures—pleural puncture, transthoracic lung biopsy and endobronchial ultrasound-guided transbronchial needle aspiration (EBUS-TBNA)—could not provide sufficient tumour material for precise tumour characterization. Only the addition of EBUS-guided transbronchial lymph node cryobiopsy (EBUS-TBLNC) enabled complete immunohistochemical and genetic tumour characterization, demonstrating PD-L1 expression in 100% of the tumour cells in the absence of actionable genetic alterations. Based on these results, immunotherapy was initiated.

## 1. Introduction

Mutational analysis and programmed death ligand 1 (PD-L1) expression are required for personalized treatment of metastatic non-small cell lung cancer (NSCLC). The detection of a targetable driver mutation has prognostic and predictive relevance, giving the patient the opportunity to receive highly effective first line targeted treatment with a kinase inhibitor [1,2,3]. The better tolerability of these oral medications in comparison to platinum-doublet chemotherapy and immunotherapy often allows for treatment of frail patients or in the context of relevant comorbidities [4,5,6]. In the absence of a targetable driver mutation or a contraindication to immunotherapy, the choice of first line treatment for patients with NSCLC depends on PD-L1 expression. In particular, for patients with a PD-L1 expression of ≥50%, Pembrolizumab or Cemiplimab monotherapy is the standard of care, while platinum-based doublet chemotherapy should be added to Pembrolizumab or Atezolizumab in cases where PD-L1 < 50%. Therefore, precise molecular and immunhistochemical characterization of metastatic NSCLC should be pursued with maximal effort [7,8].

Tissue sampling is affected by size and/or localization of tumour lesions, which may result in insufficient biopsy samples, both in terms of quantity and quality. In cases of mediastinal or hilar lymph node involvement, cytological specimens obtained by EBUS-TBNA can often confirm malignancy [9], but cytological samples with low cellularity may not always be suitable for precise immunocytochemical and molecular tumour characterization [10].

Endobronchial and transbronchial cryobiopsy can yield large tissue samples allowing exact tumour characterization and may be superior to other biopsy techniques [11]. EBUS-TBLNC is a technically challenging diagnostic procedure, which requires extensive endoscopic expertise and has only been reported in individual cases and first case series [12,13,14].

We describe the case of a patient in whom several attempts of characterization of a lung tumour by different sampling techniques (pleural puncture, transthoracic lung biopsy, EBUS-TBNA) were unsuccessful due to low cellularity of the samples. Only EBUS-TBLNC yielded sufficient tumour material for detailed immunohistochemical and molecular tumour characterization and allowed to determine the optimal systemic therapy for this patient’s non-squamous NSCLC.

## 2. Case Report

**Figure 1:**
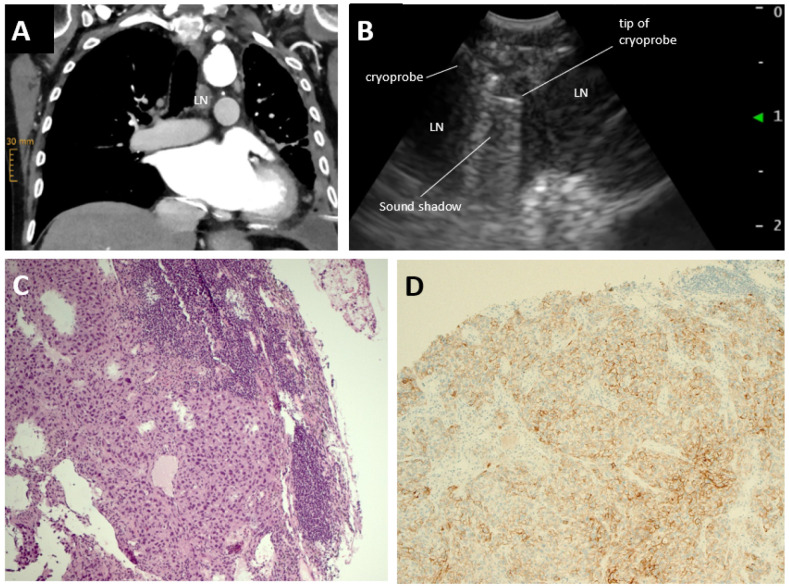
(**A**)—Coronal CT view of enlarged lymph node (LN) in LN position 4L; (**B**)—Endobronchial ultrasound showing lymph nodes (LN) with introduced cryoprobe. During the freezing process, the tip of the cryoprobe causes a sound shadow; (**C**)—Image of a EBUS-TBLNC sample showing infiltrates of non-squamous non-small cell lung carcinoma in lymph node tissue; (**D**)—Programmed death ligand 1 (PD-L1) immunohistochemistry showing strong membranous staining in 100% of tumour cells (Ventana SP263 assay, F. Hoffmann-La Roche Ltd., Basel, Switzerland).

In an 83-year-old male former smoker (30 pack-years) with multiple comorbidities but with a good performance status (ECOG 1), the work-up of a symptomatic left pleural effusion led to the detection of a lung lesion in the left lower lobe accompanied by lymphangiosis, as well as discrete hilar and mediastinal lymphadenopathy. Drainage of the pleural effusion revealed single cells of a thyroid transcription factor 1 (TTF-1)-positive pulmonary adenocarcinoma. Further molecular analysis was not possible due to the scarcity of tumour cells in the cyto spin. For further classification of the non-squamous NSCLC, cytological material was obtained by EBUS-EBNA from mediastinal lymph nodes (LN) in position 4L, the best accessible site according to the staging CT (Figure 1A). Despite representative material according to rapid on-site evaluation of four TBNAs (each with 8–10 needle passes), no malignant cells were evident in the LN aspirate. A subsequent CT guided transthoracic biopsy again yielded too few tumour cells for DNA and RNA extraction for next generation sequencing (NGS). Due to the cardiovascular comorbidities in our patient, the perioperative risk of mediastinoscopy was considered to be increased, albeit low overall, and the patient refused the intervention. Liquid biopsy was deemed unlikely to help in the detection or exclusion of actionable molecular alterations due to the overall low systemic tumour load. 

Therefore, we suggested tissue sampling with the aim of extracting solid tissue from the mediastinal LN by EBUS-TBNLC. The patient was informed about potential side effects and the limited experience with this technique. He agreed to undergo the procedure.

Under EBUS guidance, tunnelling through the bronchial wall in the direction towards LN 4L was made using a monopolar electric needle knife (Figure 1A). A 1.1 mm cryoprobe was introduced via the incisions into the LN (Figure 1B). After freezing for 7 s, several biopsies were extracted in succession, with a combined size of 0.8 × 0.7 × 0.4 cm. Neither bleeding nor any other acute or delayed periinterventional complications occurred.

Extensive infiltrates of TTF1 positive adenocarcinoma cells were seen (Figure 1C). Molecular characterization by NGS, immunohistochemistry and FISH analysis revealed a pathogenic TP53 mutation in exon 8, but no potentially targetable genetic alteration. However, tumour cells showed 100% reactivity to PD-L1 by Ventana SP263 assay (F. Hoffmann-La Roche Ltd., Basel, Switzerland) (Figure 1D).

Based on these findings and the positive risk-benefit ratio, first-line PD-1-directed therapy was initiated with Pembrolizumab as monotherapy [15,16,17]. Unfortunately, intercurrent medical issues not related to the tumour or newly initiated treatment lead to repeated hospitalizations. When the general condition of the patient declined to ECOG PS3 the decision was made with the family to discontinue palliative immunotherapy. The patient died due to complications of tumour progression five months after the first and only dose of Pembrolizumab. It is not known whether initially he responded to the treatment as no further tumour assessments were performed.

## 3. Discussion

We present a case in which, after several unsuccessful sampling attempts for NSCLC characterization, EBUS-TBLNC succeeded in adequate tumour sampling for molecular characterization, thereby allowing personalized treatment of the patient. EBUS-TBNLC may contribute to optimized molecular workup of NSCLC in the future, thereby providing important information for patient treatment.

## Data Availability

The datasets generated and analysed for this manuscript are not publicly available, but may be available from the corresponding author on reasonable request.
